# Archiatrorum Cæsareorum quondam comitis, &c. &c. constitutiones epidemicæ et morbi potissimum Lugduni Batavorum observati ex ejusdem adversariis

**Published:** 1783

**Authors:** 


III. Ger. *L. B.* Van Swieten *Archiatrorum Cæsareorum quondam comitis*, &c. &c. *constitutiones epidemicæ et morbi potissimum Lugduni Batavorum observati ex ejusdem adversariis edidit* Maximilianus Stöll, S. C. R. A. *Maj. Cons. et Prof. Med. Pract. in Universit. Vindobonensi P. O.* 8vo. Tom. 2. Vindobonæ, 1782. Tom. i, p. 463, Tom. ii, p. 424.

WE meet with nothing very interesting in this posthumous publication, which is compiled from a diary, begun by Baron Van Swieten in the year 1727 and continued till 1750.

The manner in which he kept this journal was as follows: he began with marking the name of the patient in short-hand characters, and, after adding the name of the disease, if he had determined it, or a particular mark if he was doubtful what to call it, noted the day of the month and of the disease in the margin. Each page was divided into two columns; one of these he allotted to the history of the disease, and the other was reserved for future additions, or remarks.

The diseases he has described are chiefly bilious fevers, which often had a tendency to become putrid. In these he had recourse to acids and tamarinds, but seldom to emetics. In cases of pleurisy, instead of applying a blister to the side, he prescribed a fomentation of milk and soap. The apthæ, which in some parts of his journal he ascribes to the particular constitution of the season, climate, or genius of the epidemic, were probably rather owing to his neglect of vomiting and cleansing the first passages.

It is remarkable how seldom and how sparingly he prescribed the bark. He preferred neutral salts, tamarinds, *pilul. Ruffi & cocchiæ*, and bitters to this celebrated drug even in autumnal intermittents. In tertians he often administered a purge six hours before the fit. To lying-in women he was fond of giving opium a few hours after delivery, and on the tenth day a purge of manna and Glauber's salt with lemon juice.— In the first stage of the small pox he almost always chose to take away a few ounces of blood. Such was his general plan of practice in the cases recorded in these volumes. Of many of these cases the event is not mentioned, and where they terminated fatally, it is very seldom that we meet with any account of the appearances on dissection. The following quotations will serve as a specimen of the work.

"Con-

" Conſtitutio Anni 1736.

" Julius. Ægrorum numerus admodum exi-

" guus.

" *Tertianæ*; 10 curavi; 9 adultos, juniorem

" unicum.

" *Febres acutæ continuæ*: 4 habui curandos,

" minores binos, adultos binos, ex quibus unus

" periit.

" *Dyſenterias*: duas curavi.

" *Choleras*; binas habui curandas in junio-
" ribus."

" *Febricula intermittens*.

" *Jul.* 20. Incepit die 17 parum languere, et

" febricitare, dicunt, puſtulas quaſdam illam

" habuiſſe, heri ſe bene habuerat, ſed circa

" veſperam febricitaverat leviter, ſitis magna,

" ſtrumæ in collo, dedi nitr. ſyr. fl. violar. ſucc.

" citr. &c.

" 21. Melius ſe habet, dormivit hac nocte

" bene, ſudavit, ſtrumæ in collo, non febricitat

" jam, dedi ſimilia.

" 22. Poſt meridiem febricitavit, nunc me-

" lius ſe habet, incipit parum appetere, ſudat

" de nocte, dedi ſal polychr. ſyr. fumar. aq.

" meliſſ.

" 23. Bene ſe habet ſatis, ſed ſcrophulæ ma-

" nent in collo, dedi rad. contrayerv. ℈ij ſal ab-

T 2 " ſinth.

" finth. gr. 40. M. f. pulvis infund. hydro-
" meliti."

